# Medical Therapy Algorithm With Telehealth Reduces Procedures and Transfers for Peritonsillar Abscess

**DOI:** 10.1002/lary.70136

**Published:** 2025-09-13

**Authors:** Aviv Spillinger, Erin Dimon, Anna Schmidt, Joshua Gauger, Hani I. Kuttab, David O. Francis, Sandra Y. Lin

**Affiliations:** ^1^ Department of Otolaryngology‐Head & Neck Surgery University of Wisconsin School of Medicine & Public Health Madison Wisconsin USA; ^2^ BerbeeWalsh Department of Emergency Medicine University of Wisconsin School of Medicine & Public Health Madison Wisconsin USA

**Keywords:** peritonsillar abscess, telemedicine, unnecessary procedures

## 1 Introduction

Peritonsillar abscess (PTA) is the most common deep neck space infection with ~45,000 annual cases in the United States and treatment costs exceeding $150 million USD [1]. Standard management typically involves surgical drainage by needle aspiration or incision and drainage [2]. However, emerging evidence suggests that many uncomplicated PTAs can be managed successfully with medical therapy alone [1, 3, 4, 5, 6, 7, 8, 9]. Several centers have adopted primary medical treatment algorithms, demonstrating reduced costs and minimizing overtreatment [1, 3]. This report presents preliminary findings from a quality improvement project evaluating whether a medical therapy algorithm, combined with close telehealth follow‐up, can safely reduce unnecessary procedures and inter‐hospital transfers for patients with small, uncomplicated PTAs.

## 2 Methods

This study was designated a quality improvement project by the Institutional Review Board (ID: 2023‐0874). A PTA medical therapy algorithm was developed by a multidisciplinary workgroup of emergency medicine physicians, otolaryngologists, and pharmacists based on evidence from prior publications [1, 4, 10] and implemented at a tertiary academic referral center beginning February 2024. Eligible patients presenting to an affiliated emergency department (ED) or requesting transfer from non‐affiliated hospitals were managed according to this algorithm. At affiliated hospitals, the algorithm was embedded in the electronic record, allowing ED providers to initiate medical management without otolaryngology consultation. All transfer requests from non‐affiliated hospitals or from affiliated hospitals without otolaryngology services were reviewed by the on‐call attending otolaryngologists at the tertiary center for potential enrollment.

**FIGURE 1:**
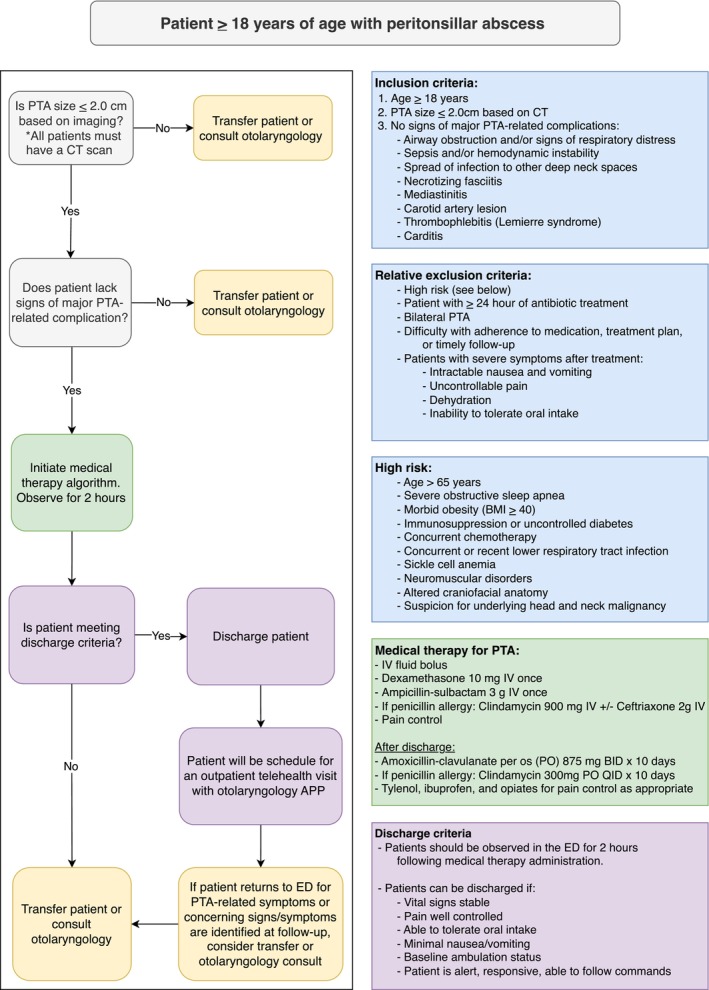
PTA medical therapy algorithm. * is to emphasize that this is an important inclusion criterea. All patients must have a CT scan to be eligble for inclusion. APP, advanced practice provider; BMI, body mass index; CT, computed tomography; IV, intravenous; PO, per os; PTA, peritonsillar abscess. [Color figure can be viewed in the online issue, which is available at www.laryngoscope.com]

The algorithm (Figure 1) applied to otherwise healthy adults with new‐onset (i.e., not previously treated) PTA presenting with mild to moderate symptoms and abscess size ≤ 2.0 cm on computed tomography (CT) imaging. A 2 cm cutoff was selected based on prior literature [5, 7] and was agreed upon by the multidisciplinary workgroup as a pragmatic balance between patient safety—by restricting eligibility to lower risk, smaller abscesses—and provider acceptance, by choosing a threshold comfortable for clinicians accustomed to managing nearly all PTAs surgically.

Discharged patients were scheduled for a phone or video telehealth follow‐up appointment with an otolaryngology Advanced Practice Provider at the tertiary referral center, ideally within 48 h. Patients also received standardized discharge instructions outlining return precautions and clinic contact information. Outcomes were assessed through follow‐up documentation or, if unavailable, by chart review. Treatment failure was defined as undergoing a salvage procedure (e.g., needle aspiration, incision and drainage) or developing a major PTA‐related complication (e.g., airway obstruction, mediastinitis) within 30 days of initial presentation.

## 3 Results

Forty‐eight patients were managed according to the algorithm (median age: 35 years; interquartile range: 26–49.5; 50% female). Thirty‐seven patients (77.1%) presented to affiliated hospitals and 11 (22.9%) to non‐affiliated hospitals. Notably, 27 patients (56.3%) presented to hospitals without otolaryngology services, which—prior to the algorithm's implementation—would have traditionally necessitated transfer for surgical drainage. The average distance from non‐affiliated hospitals to the tertiary center was 48.4 miles (range: 22.3–85.2 miles).

Medical therapy alone was successful for 43 (89.6%) patients. No major PTA‐related complications occurred. Five patients (10.4%) experienced treatment failures: four underwent salvage incision and drainage within 48 h of initial presentation, and two required transfers from hospitals without otolaryngology services. Therefore, 25 of 27 patients (92.6%) presenting to hospitals without otolaryngology services were successfully medically managed locally without requiring transfer. Telehealth follow‐up was completed for 28 patients (58.3%), with 16 (33.3%) patients completing follow‐up within 48 h.

## 4 Discussion

Consistent with prior studies, this report suggests that surgical drainage can often be safely avoided for small (≤ 2 cm), uncomplicated PTAs, potentially reducing morbidity and healthcare costs of inter‐hospital transfers [1, 3, 4, 5, 6, 7]. Southern California Kaiser Permanente Medical Group reported thousands of medically managed PTA cases, citing an 8.1% treatment failure rate [1]. In the present study, the medical treatment failure rate was 10.4%, and inter‐hospital transfers were avoided for 92.6% of patients presenting to hospitals without otolaryngology services. Prior to implementation, our institution typically accepted most transfer requests from hospitals without otolaryngology coverage for surgical drainage of PTAs, including those with small (< 2 cm) abscesses. Although historical transfer rates for small PTAs were not captured and cannot be quantified retrospectively, our findings suggest that the algorithm enabled many of these patients to be safely managed locally without transfer.

To the authors' knowledge, this is the first PTA medical therapy algorithm to incorporate telehealth follow‐up. Telehealth appointments were used to evaluate treatment response, provide further education, and counsel patients on return precautions and signs of treatment failure (e.g., worsening throat or neck pain, worsening trismus, respiratory distress). Additional advantages of telehealth included improved clinic resource utilization and increased convenience for patients, particularly those referred from non‐local hospitals. Although standardized discharge instructions were provided to all patients, scheduled telehealth follow‐up served as an added safeguard by enabling timely reassessment and early recognition of treatment failure.

This report describes preliminary findings from an ongoing prospective quality improvement study, with several limitations. Although standardized inclusion criteria were established, there was variability in decision‐making across providers, and patients from outside non‐affiliated centers were eligible only if the on‐call otolaryngology attending was contacted. Data on transfer calls, excluded patients, and reasons for non‐enrollment were not systematically available.

## 5 Conclusion

A medical therapy algorithm with close telehealth follow‐up may safely reduce unnecessary procedures and inter‐hospital transfers for small, uncomplicated PTAs. Further patient enrollment will be important to confirm the safety and efficacy of the PTA medical therapy algorithm.

## Disclosure

The authors have nothing to report.

## Conflicts of Interest

The authors declare no conflicts of interest.

## Data Availability

The data that support the findings of this study are available from the corresponding author upon reasonable request.
